# SparkBWA: Speeding Up the Alignment of High-Throughput DNA Sequencing Data

**DOI:** 10.1371/journal.pone.0155461

**Published:** 2016-05-16

**Authors:** José M. Abuín, Juan C. Pichel, Tomás F. Pena, Jorge Amigo

**Affiliations:** 1 Centro de Investigación en Tecnoloxías da Información (CITIUS), Universidade de Santiago de Compostela, Santiago de Compostela, Spain; 2 Fundación Pública Galega de Medicina Xenómica (SERGAS), Santiago de Compostela, Spain; 3 Grupo Medicina Xenómica, Instituto de Investigación Sanitaria de Santiago de Compostela, Santiago de Compostela, Spain; University of Helsinki, FINLAND

## Abstract

Next-generation sequencing (NGS) technologies have led to a huge amount of genomic data that need to be analyzed and interpreted. This fact has a huge impact on the DNA sequence alignment process, which nowadays requires the mapping of billions of small DNA sequences onto a reference genome. In this way, sequence alignment remains the most time-consuming stage in the sequence analysis workflow. To deal with this issue, state of the art aligners take advantage of parallelization strategies. However, the existent solutions show limited scalability and have a complex implementation. In this work we introduce SparkBWA, a new tool that exploits the capabilities of a big data technology as Spark to boost the performance of one of the most widely adopted aligner, the Burrows-Wheeler Aligner (BWA). The design of SparkBWA uses two independent software layers in such a way that no modifications to the original BWA source code are required, which assures its compatibility with any BWA version (future or legacy). SparkBWA is evaluated in different scenarios showing noticeable results in terms of performance and scalability. A comparison to other parallel BWA-based aligners validates the benefits of our approach. Finally, an intuitive and flexible API is provided to NGS professionals in order to facilitate the acceptance and adoption of the new tool. The source code of the software described in this paper is publicly available at https://github.com/citiususc/SparkBWA, with a GPL3 license.

## 1 Introduction

The history of modern DNA sequencing starts more than thirty-five years ago. These years have seen amazing growth in DNA sequencing capacity and speed, especially after the appearance of next-generation sequencing (NGS) and massive parallel sequencing in general. NGS has led to an unparalleled explosion in the amount of sequencing data available. For instance, new sequencing technologies, such as Illumina HiSeqX^™^ Ten, generate up to 6 billion sequence reads per run. Mapping these data onto a reference genome is often the first step in the sequence analysis workflow. This process is very time-consuming and, although state-of-art aligners were developed to efficiently deal with large amount of DNA sequences, the alignment process still remains a bottleneck in bioinformatics analyses. In addition, NGS platforms are evolving very quickly, pushing the sequencing capacity to unprecedented levels.

To address this challenge we propose to take advantage of parallel architectures using big data technologies in order to boost performance and improve scalability of the sequence aligners. In this way, it will be possible to process huge amounts of sequencing data within a reasonable time. In particular, Apache Spark [1] has been considered as the big data framework in this work. Spark is a cluster computing framework which supports both in-memory and on-disk computations in a fault tolerant manner using distributed memory abstractions known as Resilient Distributed Datasets (RDDs). An RDD can be explicitly cached in memory across cluster nodes and reused in multiple MapReduce-like parallel operations.

In this paper we introduce SparkBWA, a new tool that integrates the Burrows-Wheeler aligner (BWA) [2] into the Spark framework. BWA is one of the most widely used alignment tools for mapping sequence reads to a large reference genome. It consists of three different algorithms for aligning short reads. SparkBWA was designed to meet three requirements. First, SparkBWA should outperform BWA and other BWA-based aligners both in terms of performance and scalability. Note that BWA has its own parallel implementation for shared-memory systems. The second requirement is related to keep the compatibility of SparkBWA with future and legacy versions of BWA. Since BWA is constantly evolving to include new functionalities and algorithms, it is important for SparkBWA to be agnostic regarding the BWA version. This is an important difference with respect to other existent tools based on BWA, which require modifications of the BWA source code. Finally, NGS professionals demand solutions to perform sequence alignments efficiently in such a way that the implementation details are completely hidden to them. For this reason SparkBWA provides a simple and flexible API to handle all the aspects related to the alignment process. In this way, bioinformaticians only need to focus on the scientific problem to deal with.

SparkBWA has been evaluated both in terms of performance and memory consumption, and a thorough comparison between SparkBWA and several state-of-art BWA-based aligners is also provided. Those tools take advantage of different parallel approaches as Pthreads, MPI, and Hadoop to improve the performance of BWA. Performance results demonstrate the benefits of our proposal.

This work is structured as follows: Section 2 explains the background of the paper. Section 3 discusses the related work. Section 4 details the design of SparkBWA and introduces its API. Section 5 presents the experiments carried out to evaluate the behavior and performance of our proposal together with a comparison to other BWA-based tools. Finally, the main conclusions derived from the work are explained in Section 6.

## 2 Background

### 2.1 MapReduce programming model

MapReduce [3] is a programming model introduced by Google for processing and generating large data sets on a huge number of computing nodes. A MapReduce program execution is divided into two phases: *map* and *reduce*. In this model, the input and output of a MapReduce computation is a list of key-value pairs. Users only need to focus on implementing map and reduce functions. In the map phase, map workers take as input a list of key-value pairs and generate a set of intermediate output key-value pairs, which are stored in the intermediate storage (i.e., files or in-memory buffers). The reduce function processes each intermediate key and its associated list of values to produce a final dataset of key-value pairs. In this way, map workers achieve data parallelism, while reduce workers perform parallel reduction. Note that parallelization, resource management, fault tolerance and other related issues are handled by the MapReduce runtime.

Apache Hadoop [4] is the most successful open-source implementation of the MapReduce programming model. Hadoop consists, basically, of three layers: a data storage layer (HDFS—Hadoop Distributed File System [5]), a resource manager layer (YARN—Yet Another Resource Negociator [6]), and a data processing layer (Hadoop MapReduce Framework). HDFS is a block-oriented file system based on the idea that the most efficient data processing pattern is a write-once, read-many-times pattern. For this reason, Hadoop shows good performance with embarrassingly parallel applications requiring a single MapReduce execution (assuming intermediate results between map and reduce phases are not huge), and even for applications requiring a small number of sequential MapReduce executions [7]. Note that Hadoop can also efficiently handle jobs composed by one or more map functions by chaining several mappers followed by a reducer function and, optionally, zero or more map functions, saving the disk I/O cost between map phases. For more complex workflows, solutions as Apache Oozie [8] or Cascading [9], among others, should be used.

The main disadvantage of these workflow managers is the loss of performance when HDFS has to be used to store intermediate data. For example, an iterative algorithm can be expressed as a sequence of multiple MapReduce jobs. Since different MapReduce jobs cannot shared data directly, intermediate results have to be written to disk and read again from HDFS at the beginning of the next iteration, with the consequent reduction in performance. It is worth noting that even each iteration of the algorithm could consist of one or several MapReduce executions. In this case, the degradation in terms of performance is even more noticeable.

### 2.2 Apache Spark

Apache Spark is a cluster computing framework designed to overcome the Hadoop limitations in order to support iterative jobs and interactive analytics, originally developed at University of California, Berkeley [1], now managed under the umbrella of the Apache Software Foundation. Spark uses a master/slave architecture with one central coordinator (driver) and many distributed workers (executors). It supports both in-memory and on-disk computations in a fault tolerant manner by introducing the idea of Resilient Distributed Datasets (RDDs) [10]. An RDD represents a read-only collection of objects partitioned across the cluster nodes that can be rebuilt if a partition is lost. Users can explicitly cache an RDD in memory across machines and reuse it in multiple MapReduce-like parallel operations. By using RDDs, programmers can perform iterative operations on their data without writing intermediary results to disk. In this way, Spark is well-suited, for example, to machine learning algorithms.

RDDs can be created by distributing a collection of objects (e.g., a list or set) or by loading an external dataset from any storage source supported by Hadoop, including the local file system, HDFS, Cassandra [11], HBase [12], Parquet [13], etc. On created RDDs, Spark supports two types of parallel operations: transformations and actions. Transformations are operations on RDDs that return a new RDD, such as map, filter, join, groupByKey, etc. The resulting RDD will be stored in memory by default, but Spark also supports the option of writing RDDs to disk whenever necessary. On the other hand, actions are operations that kick off a computation, returning a result to the driver program or writing it to storage. Examples are collect, count, take, etc. Note that transformations on RDDs are lazily evaluated, meaning that Spark will not begin to execute until it sees an action.

A Spark application, at a high level, consists of a *driver* program which contains the application’s main function and defines RDDs on the cluster, then applies transformations and actions to them. A Spark program implicitly creates, from defined transformations and actions over RDDs, a logical directed acyclic graph (DAG) of operations, which is converted by the driver into a physical execution plan. This plan is then optimized, e.g., merging several map transformations, and individual tasks are bundled up and prepared to be sent to the cluster. The driver connects to the cluster through a SparkContext. An *executor* or worker process is in charge of effectively running the tasks on each node of the cluster.

Apache Spark provides both Python and Scala interactive shells, which let the user interact with data that is distributed on disk or in memory across many machines. Apart from running interactively, Spark can also be linked into applications in either Java, Scala, or Python. Finally, we must highlight that Spark can run in local mode, in standalone mode on a cluster, or using a cluster manager such as Mesos [14] or YARN [6].

### 2.3 Burrows-Wheeler aligner (BWA)

Burrows-Wheeler aligner (BWA) is a very popular open-source software for mapping sequence reads to a large reference genome. In particular, it consists of three different algorithms: BWA-backtrack [2], BWA-SW [15] and BWA-MEM [16]. The first algorithm is designed for short Illumina sequence reads up to 100bp (base pairs), while the others are focused on longer reads. BWA-MEM, which is the latest, is preferred over BWA-SW for 70bp or longer reads as it is faster and more accurate. In addition, BWA-MEM has shown better performance than other several state-of-art read aligners for mapping 100bp or longer reads.

As we have previously noted, sequence alignment is a very time-consuming process. For this reason BWA has its own parallel implementation, but it only supports shared memory machines. Therefore, scalability is limited by the number of threads (cores) and memory available in just one computing node.

**Fig 1:**
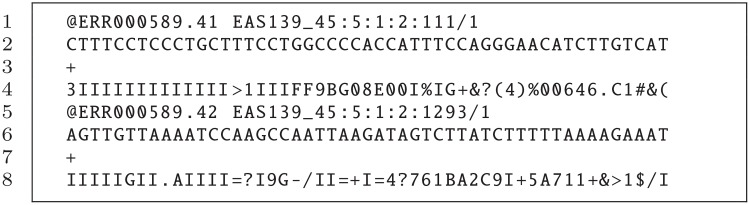
FASTQ file format example.

Although BWA can read unaligned BAM [17] files, it typically accepts FASTQ format [18] as input, which is one of the most common output formats for raw sequence reads. It is a plain text format in such a way that every four lines describe a sequence or read. An example including two reads is shown in Fig 1. The information provided per read is: identifier (first line), sequence (second line), and the quality score of the read (fourth line). An extra field, represented by symbol ‘+’, is used as separator between the data and the quality information (third line). BWA is able to use single-end reads (one input FASTQ file) and paired-end reads (two input FASTQ files). When considering paired-end reads, two sequences corresponding to both ends of the same DNA fragment are available. Both reads are included in different input files using the same identifier and in the same relative location within the files. In this way, considering our example, the corresponding pair of sequence #2 will be located in line 5 of the other input file. On the other hand, the output of BWA is a SAM (Sequence Alignment/Map) [17] file, which is the standard format for storing read alignments against reference sequences. This SAM file will be further required, for example, for performing variant discovery analysis.

## 3 Related Work

We can find in the literature several interesting tools based on the Burrows-Wheeler aligner which exploit parallel and distributed architectures to increase the BWA performance. Some of these works are focused on big data technologies like SparkBWA, but they are all based on Hadoop. Examples are BigBWA [19], Halvade [20] and SEAL [21]. BigBWA is a recent sequence alignment tool developed by the authors which shows good performance and scalability results with respect to other BWA-based approaches. Its main advantage is that it does not require any modification of the original BWA source code. This characteristic is shared by SparkBWA in such a way that both tools keep the compatibility with future and legacy BWA versions.

SEAL uses Pydoop [22], a Python implementation of the MapReduce programming model that runs on the top of Hadoop. It allows users to write their programs in Python, calling BWA methods by means of a wrapper. SEAL only works with a particular modified version of BWA. Since SEAL is based on BWA version 0.5, it does not support the new BWA-MEM algorithm for longer reads.

Halvade is also based on Hadoop. It includes a variant detection phase which is the next stage after the sequence alignment in the DNA sequencing workflow. Halvade calls BWA from the mappers as an external process which may cause timeouts during the Hadoop execution if the task timeout parameter is not adequately configured. Therefore, *a priori* knowledge about the execution time of the application is required. Note that setting the timeout parameter to high values causes problems in the detection of actual timeouts, which reduces the efficiency of the fault tolerance mechanisms of Hadoop. To overcome this issue, as it is explained in further sections, SparkBWA uses Java Native Interface (JNI) to call the BWA methods.

Another approach is applying standard parallel programming paradigms to BWA. For instance, pBWA [23] uses MPI to parallelize BWA in order to carry out the alignments on a cluster. We must highlight that pBWA lacks fault tolerant mechanisms in contrast to SparkBWA. In addition, pBWA, as well as SEAL, does not support the BWA-MEM algorithm.

Several solutions try to take advantage of the computing power of the GPUs to improve the performance of BWA. This is the case of BarraCUDA [24], which is based on the CUDA programming model. It requires the modification of the BWT (Burrows Wheeler Transform) alignment core of BWA to exploit the massive parallelism of GPUs. Unlike SparkBWA which supports all the algorithms included in BWA, BarraCUDA only supports the BWA-backtrack algorithm for short reads. It shows improvements up to 2× with respect to the threaded version of BWA. It is worth to mention that due to some changes in the BWT data structure of most recent versions of BWA, BarraCUDA is only compatible with BWTs generated with BWA versions 0.5.x. Other important sequence aligners (not based on BWA) that make use of GPUs are CUSHAW [25], SOAP3 [26] and SOAP3-dp [27].

Some researchers have focused on speeding up the alignment process using the new Intel Xeon Phi coprocessor (Intel Many Integrated Core architecture—MIC). For example, mBWA [28], which is based on BWA, implements the BWA-backtrack algorithm for the Xeon Phi coprocessor. mBWA allows to use concurrently both host CPU and coprocessor in order to perform the alignment, reaching speedups of 5× with respect to BWA. Another solution for the MIC coprocessors can be found in [29]. A third aligner that takes advantage of the MIC architecture is MICA [30]. Authors claim that it is 5× faster than threaded BWA using 6 cores. Note that, unlike SparkBWA, this tool is not based on BWA.

Another researchers exploit fine-grain parallelism in FPGAs (Field Programmable Gate Arrays) to increase the performance of several short-read aligners including some based on BWT [31–33].

Finally, a recent work uses Spark to increase the performance of one of the best well-known alignment algorithms, the Smith-Waterman algorithm [34]. Performance results demonstrate the potential of Spark as framework for this type of applications.

## 4 SparkBWA

This section introduces a new tool called SparkBWA, which integrates the Burrows-Wheeler aligner into the Spark framework. As stated in the Introduction, SparkBWA was designed with the following three objectives in mind:

- It should boost BWA and other aligners based on BWA in terms of performance and scalability.
- It should be version-agnostic regarding BWA, which assures its compatibility with future or legacy BWA versions.
- An intuitive and flexible API should be provided to NGS professionals with the aim of facilitating the acceptance and adoption of the new tool.

Next, a detailed description of the design and implementation of SparkBWA is provided, together with the specification of the high-level API.

### 4.1 System design

SparkBWA workflow consists of three main stages: RDDs creation, map, and reduce phases. In the first phase input data are prepared to feed the map phase where the alignment process is, strictly speaking, carried out. In particular, RDDs are created from the FASTQ input files, which are stored using HDFS. Note that, in this work, we assume HDFS as distributed file system. In this way, data is distributed across the computing nodes so it can be processed in parallel in the map phase. The read identifier in the FASTQ file format is used as *key* in the RDDs (see the example of Fig 1). In this way, key-value pairs generated from an input file have the following appearance <*read_id, read_content*>, where *read_content* contains all the information of the corresponding sequence with *read_id* identifier. These RDDs will be used afterwards in the map phase. This approach works properly when considering single-end reads, that is, when there is only one FASTQ input file.

**Fig 2:**
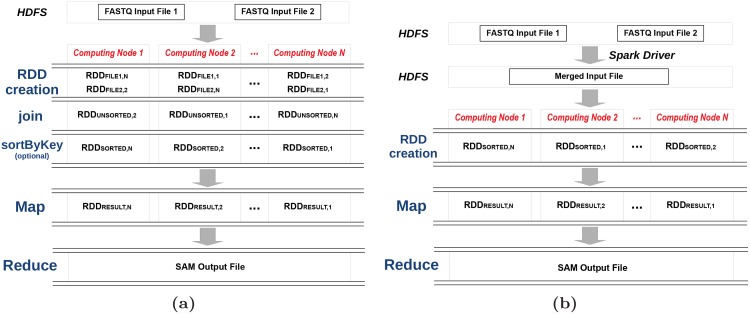
SparkBWA workflow for paired-end reads using (a) Join and (b) SortHDFS approaches.

However, SparkBWA should also support paired-end reads. In that case, two RDDs will be created, one per input file, and distributed among the nodes. Spark distributes RDDs in such a way that is not guaranteed that the *i*-th data split (partition) of both RDDs will be processed by the same mapper. In this way, a mapper cannot process paired-end reads since they are always located in the same *i*-th data partition of both RDDs. This behavior can be observed in the RDD creation stage of the example displayed in Fig 2(a). Two solutions are proposed in order to overcome this issue:

- **Join**: This approach is based on using the Spark *join* operation, which is a transformation that merges two RDDs together by grouping elements with the same key. This solution is illustrated in Fig 2(a). Since the key is the same for paired reads in both input files, the result after the join operation will be an unique RDD with the format: <*read_id, Tuple*<*read_content1, read_content2*>> (RDD_UNSORTED_ in the example). The resulting RDD after the join operation does not preserve the previous order of the reads from the FASTQ files. This is not a problem because mappers will process the paired-end reads independently from each other. However, Spark provides the *sortByKey* transformation to sort RDD records according to its key. In the example, the new RDD created after applying this operation is RDD_SORTED_. We must highlight that the sortByKey operation is expensive in terms of memory consumption. For this reason this step is optional in the SparkBWA dataflow and users should enable it specifically, if they want to get a sorted output.
- **SortHDFS**: A new approach is presented in order to avoid the *join* and *sortByKey* operations (see Fig 2(b)). This solution can be considered as a preprocessing stage which requires reading and writing to/from HDFS. In this way, FASTQ input files are accessed directly by using the HDFS Hadoop library from the Spark driver program. Paired-end reads (that is, those with the same identifier in the two files) are merged into one record in a new HDFS file. As BWA requires to distinguish between both sequences in the pair, a separator string is used to facilitate the subsequent parsing process in the mappers. Afterwards, an RDD is created from the new file (RDD_SORTED_ in the figure). In this way, key-value pairs have the following format <*read_id, merged_content*>. This solution performs several time consuming I/O operations, but saves a lot of memory in comparison to the join & sortByKey approach as we illustrate in Section 5.

Once RDDs are available, the map phase starts. Mappers will apply the sequence alignment algorithm from BWA on the RDDs. However, calling BWA from Spark is not straightforward as BWA source code is written in C language and Spark only allows to run code in Scala, Java or Python. To overcome this issue SparkBWA takes advantage of the Java Native Interface (JNI), which allows the incorporation of native code written in languages as C and C++ as well as Java code.

The map phase was designed using two independent software layers. The first one corresponds to the BWA software package, while the other is responsible to process RDDs, pass the input data to the BWA layer and collect the partial results from the map workers. We must highlight that mappers only perform calls to the BWA main function by means of JNI. This design avoids any modification of the original BWA source code, which assures the compatibility of SparkBWA with future or legacy BWA versions. In this way, our tool is version-agnostic regarding BWA. Note that this approach is similar to the one adopted in the BigBWA tool [19].

Another advantage of the two-layers design is that the alignment process could be performed using two levels of parallelism. The first level corresponds to the map processes distributed across the cluster. In the second level each individual map process is parallelized using several threads, taking advantage of the BWA parallel implementation for shared memory machines. We refer to this mode of operation as *hybrid mode*. This mode can be enabled by the user through the SparkBWA API.

On the other hand, BWA uses a reference genome as input in addition to the FASTQ files. All mappers require the complete reference genome, so it has to be shared among all computing nodes using NFS or stored locally in the same location of all the nodes (e.g., using Spark broadcast variables).

Once the map phase is complete, SparkBWA creates one output SAM file in HDFS per launched map process. Finally, users could merge all the outputs into one file choosing to execute an additional reduce phase.

### 4.2 SparkBWA API

One of the requirements of SparkBWA is to provide bioinformaticians an easy and powerful way to perform sequence alignments using a big data technology as Apache Spark. With this goal in mind a basic API is provided. It allows NGS professionals to focus only in the scientific problem, while design and implementation details of SparkBWA are completely transparent to them.

SparkBWA can be used from the Spark shell (Scala) or console. Table 1 summarizes the API methods to set the SparkBWA options in the shell together with their corresponding console arguments. For example, it is possible to choose the number of data partitions, how RDDs are created, or the number of threads used per mapper (hybrid mode).

**Table 1:** API methods and console arguments to set the SparkBWA options.

Function	Default	Console argument	Description
setUseReducer(boolean)	False	-r	Use a reducer to generate one output SAM file.
setPartitionNumber(int)	Auto	none |-partitions <num>	By default, data is split into pieces of HDFS block size. Otherwise, input data is split into num partitions.
setSortFastqReads(int)	Join	none |-sort |-sorthdfs	Set the RDDs creation approach for paired-end reads: Join (0), Join & sortByKey (1) or SortHDFS (2).
setNumThreads(int)	1	-threads <num>	If num > 1, hybrid parallelism mode is enabled in such a way that each map process is executed using *num* threads.
setAlgorithm(int)	BWA-MEM	-mem |-aln |-bwasw	Set the alignment algorithm: BWA-MEM (0), BWA-backtrack (1), BWA-SW (2)
setPairedReads(boolean)	Paired	-paired |-single	Use single-end (one FASTQ input file) or paired-end reads (two FASTQ input files).
setIndexPath(string)	–	-index <prefix>	Set the path to the reference genome (mandatory option).
setInputPath(string)	–	Positional	Set the path (in HDFS) to the FASTQ input file (mandatory option for single-end and paired-end reads).
setInputPath2(string)	–	Positional	Set the path (in HDFS) to the second FASTQ input file (mandatory option for paired-end reads).
setOutputPath(string)	–	Positional	Set the location (in HDFS) where the output SAM file/s will be stored.

**Fig 3:**
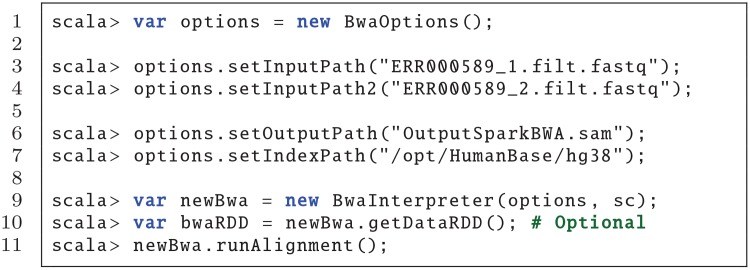
Example running SparkBWA from the Spark Shell (Scala).

**Fig 4:**
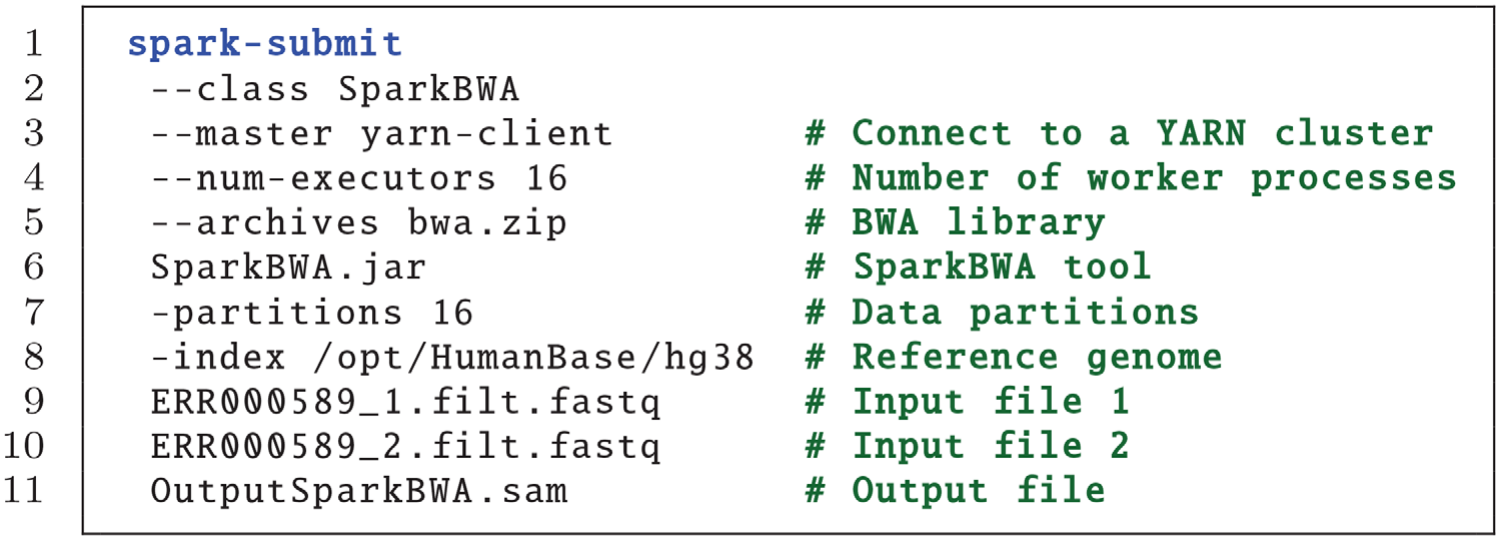
Example running SparkBWA from the console.

1. *Spark Shell*: Spark comes with an interactive shell that provides a simple way to learn the Spark API, as well as a powerful tool to analyze data interactively. It is available in either Scala (which runs on the Java VM and is thus a good way to use existing Java libraries) or Python. Current SparkBWA version only supports the Scala shell.
  An example of how to perform an alignment using SparkBWA from the Spark shell is illustrated in Fig 3. First, the user should create a BwaOptions object to specify the options desired in order to execute SparkBWA (line 1). In this example only the mandatory options are set (lines 3–7). Refer to Table 1 for additional options.
  Once the options are specified, a new BwaInterpreter should be created (line 9). At that moment RDDs are created from the input files according to the implementation detailed previously in Section 4.1. It is worth to mention that the RDDs creation is lazy evaluated, which means that Spark will not begin to execute until an action is called. This action could be, for example, obtaining explicitly the input RDD using the getDataRDD method (line 10). This method is very useful in the sense that it allows the users to apply to the input RDDs all the transformations and actions that the Spark API provides in addition to user-defined functions. Note that using the getDataRDD method is not necessary to perform the sequence alignment with SparkBWA. Another action that triggers the RDDs creation is runAlignment, which will execute the complete SparkBWA workflow including the map and reduce phases (line 11).
2. *Console*: It is also possible to run SparkBWA from the console, that is, using the spark-submit command. An example is shown in Fig 4. spark-submit provides a variety of options that let the user control specific details about a particular run of an application (lines 2–6). In our case, the user also needs to pass as arguments the SparkBWA options to Spark (lines 7–11). All the flags supported by SparkBWA are detailed in Table 1.

Therefore, SparkBWA provides an easy and flexible interface in such way that users could perform a sequence alignment writing just a couple of lines of code in the Spark shell, or using the standard spark-submit tool from the console.

## 5 Evaluation

In this section SparkBWA is evaluated in terms of performance, scalability, and memory consumption. First, a complete description of the experimental setup is provided. Next, SparkBWA is analyzed in detail paying special attention to the creation of RDDs and its different modes of operation (regular and hybrid). Finally, in order to validate our proposal, a comparison to several BWA-based aligners is also provided.

### 5.1 Experimental Setup

SparkBWA was tested using data from the 1000 Genomes Project [35]. The main characteristics of the input datasets are shown in Table 2. Number of reads refers to the number of sequences to be aligned to the reference genome. The read length is expressed in terms of the number of base pairs (bp).

**Table 2:** Main characteristics of the input datasets from the 1000 Genomes Project.

Tag	Name	Number of reads	Read length (bp)	Size (GiB)
D1	NA12750/ERR000589	12×10^6^	51	3.4
D2	HG00096/SRR062634	24.1×10^6^	100	11.8
D3	150140/SRR642648	98.8×10^6^	100	48.3

As the alignment can be performed for single or paired-ended reads, it is needed to determine which one is going to be used during the evaluation. As the paired-ended DNA sequencing reads provide superior alignment across DNA regions containing repetitive sequences reads, it is the one that is considered in this work. In this way, each dataset consists of two FASTQ files.

Experiments were carried out on a six-node cluster. Each node consists of four AMD Opteron 6262HE processsors (4×16 cores) with 256 GiB of memory (i.e., 4 GiB per core). Nodes are connected through a 10GbE network. The Hadoop and Spark versions used are 2.7.1 and 1.5.2, respectively, running on a CentOS 6.7 platform. OpenMPI 4.4.7 was used in the experiments that require MPI. The cluster was configured assigning about 11 GiB of memory per YARN container (map and reduce processes) in such a way that a maximum of 22 containers per node can be executed concurrently. This memory configuration allows each SparkBWA container to execute one BWA process, including the memory required to store the reference genome index. Note that the master node in the cluster is also used as computing node.

The behavior of SparkBWA is compared to several state of the art BWA-based aligners. In particular, we have considered the tools detailed in Table 3. A brief description of these tools is provided in Section 3. pBWA and SEAL only support the BWA-backtrack algorithm because both are based on BWA version 0.5 (2009). For fair comparison with these tools, SparkBWA obtains its performance results for the BWA-backtrack algorithm also using BWA version 0.5. In the case of BWA-MEM, three different aligners are evaluated: BigBWA, Halvade and BWA (shared-memory threaded version). For the BWA-MEM performance evaluation, the latest available BWA version at the moment of writing the paper is used (version 0.7.12, December 2014). We must highlight that all the time results shown in this section were calculated as the average value (arithmetic mean) of twenty executions.

**Table 3:** Algorithms and BWA-based aligners evaluated.

Algorithm	Tools	Parallelization Technology
BWA-backtrack	pBWA [23]	MPI
	SEAL [21]	Hadoop
	SparkBWA	Spark
BWA-MEM	BWA [16]	Pthreads
	BigBWA [19]	Hadoop
	Halvade [20]	Hadoop
	SparkBWA	Spark

### 5.2 Performance Evaluation

#### 5.2.1 RDDs creation

**Fig 5:**
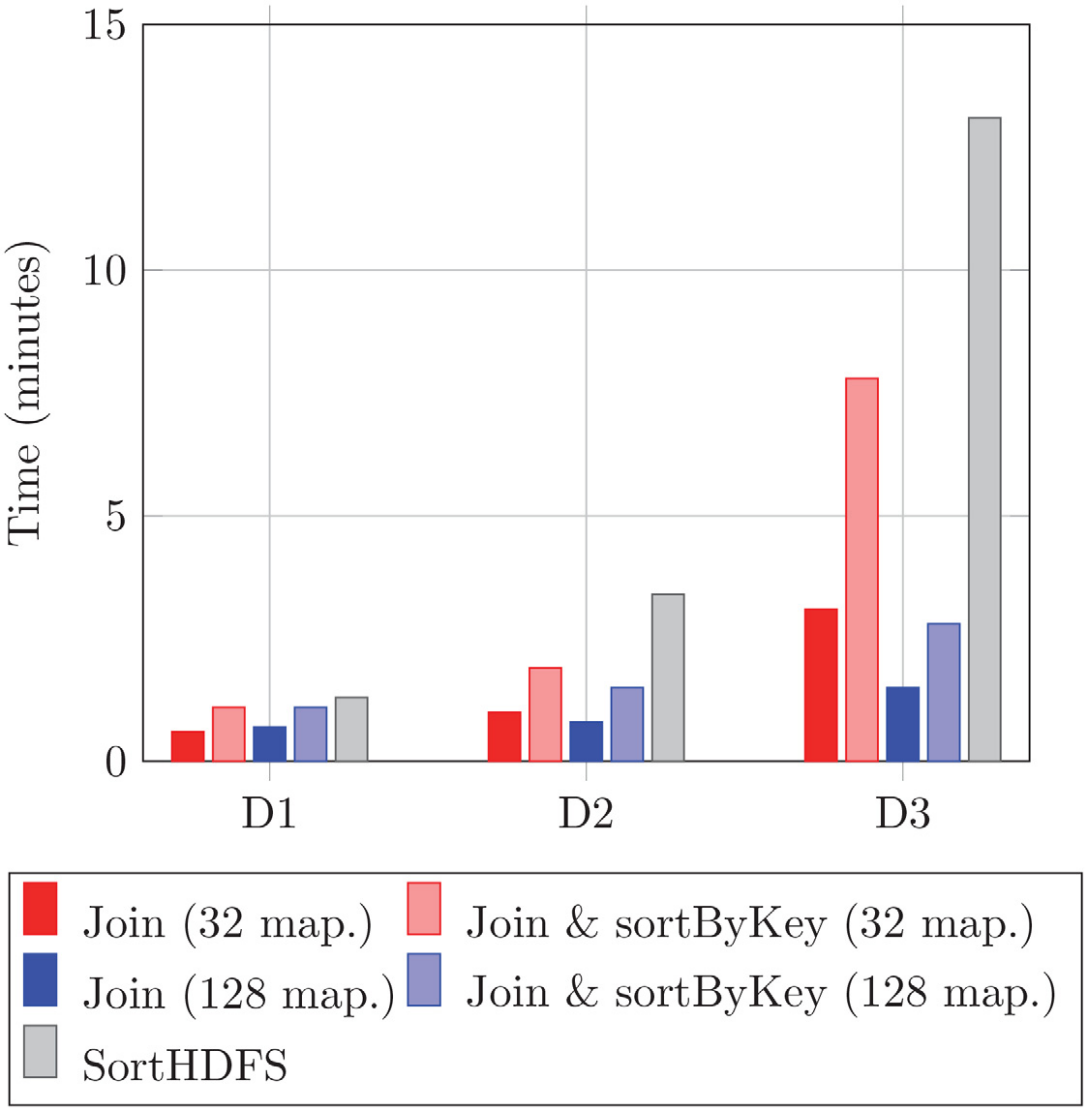
Overhead of the RDDs sorting operation considering different datasets.

The first stage in the SparkBWA workflow is the creation of the RDDs, which can include a sorting phase (see Section 4.1). Two different approaches were considered to implement this phase: Join and SortHDFS. The first one is based on the Spark *join* operation, and includes an additional optional step to sort the input paired-end reads by key (*sortByKey* operation). The latter approach requires reading and writing to/from HDFS. As we pointed out previously, this solution can be considered as a preprocessing stage. Both solutions have been evaluated in terms of the overhead considering different datasets. Results are displayed in Fig 5.

The performance of the Join approach (with and without the *sortByKey* transformation) depends on the number of map processes, so this operation was evaluated using 32 and 128 mappers. As the number of mappers increases, the sorting time improves because the size of the data splits computed by each worker is smaller. This behavior was observed for all the datasets, especially when D3 is considered.

The overhead for all the approaches, as it was expected, increases with the size of the dataset. However, the increment rate is higher for SortHDFS. For example, sorting D3 is 10× slower than sorting D1, while the Join approach with and without *sortByKey* is at most only 5× and 7× slower respectively. Note that D3 is more than 14× bigger than D1 (see Table 2).

The Join approach is always better in terms of overhead, especially as the number of map processes increases. For example, sorting D3 takes only 1.5 minutes with 128 mappers (*join* only), which means a speedup of 8.7× with respect to SortHDFS. It can also be observed that sorting the RDDs by key consumes extra time. In particular, the overhead means on average doubling the time required by the sorting process when only the *join* transformation is performed.

**Fig 6:**
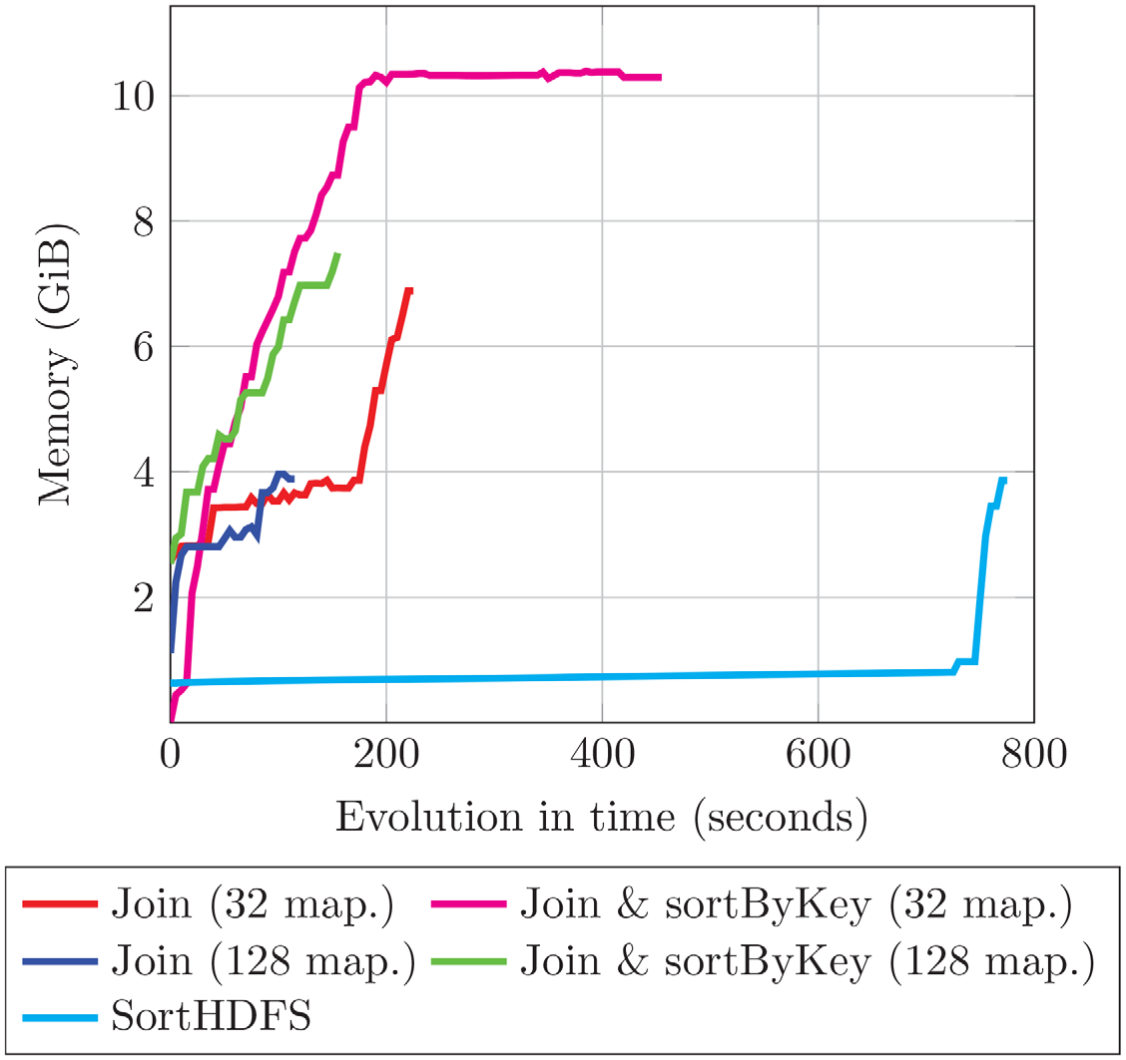
Memory consumed by SparkBWA during the RDDs sorting operation when considering dataset D3.

On the other hand, speed is not the only parameter that should be taken into account when performing the RDDs sorting. In this way, memory consumption has also been analyzed. In order to illustrate the behavior of both sorting approaches we have considered D3 as dataset. Fig 6 shows the memory used by a map process during the sorting operation period.

According to the results, the Join approach always consumes more memory than SortHDFS. This is caused by the *join* and *sortByKey* Spark operations on the RDDs, which both are in-memory transformations. It is especially relevant the differences observed when the elements of the RDDs are sorted by key with respect to applying only the *join* operation. In this way, the *sortByKey* operation consumes about 3 GiB extra per mapper for this dataset, which means increasing more than 30% the memory required by SparkBWA in this phase. Note that when considering 32 workers the maximum memory available per container is reached. The memory used by 128 workers is lower because RDDs are split into smaller pieces with respect to considering 32 workers. On the other hand, SortHDFS requires a maximum of 4 GiB to preprocess the dataset in the example. In this way, SortHDFS is the best choice if the memory resources are limited or not enough to perform the Join operation (with or without *sortByKey*). Note that the overall behavior illustrated in Fig 6 agrees with the observations for the other datasets.

#### 5.2.2 Hybrid mode

As stated in Section 4.1, the design of SparkBWA in two software layers allows to use several threads per worker in such a way that the alignment process is performed taking advantage of two levels of parallelism. In this way, SparkBWA has two modes of operation: regular and hybrid. The hybrid mode refers to using more than one thread per map process, while the regular behavior executes each mapper sequentially.

**Fig 7:**
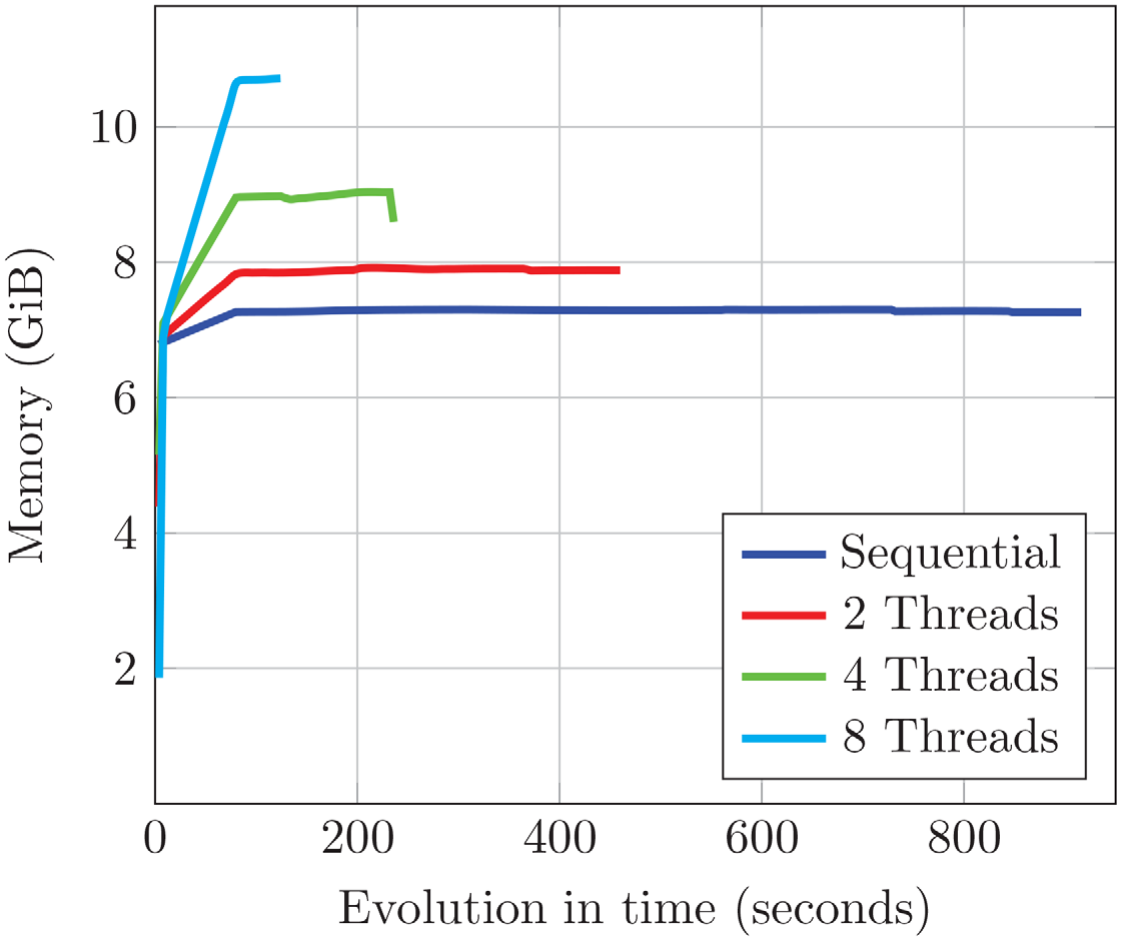
Memory consumed by a worker process executing the BWA-MEM algorithm with different threads.

The memory used by each mapper when hybrid mode is enabled increases with the number of threads involved in the computation. However, since the index reference genome required by BWA is shared among threads, this increase is moderate. This behavior is illustrated in Fig 7, where BWA-MEM is executed using different number of threads with a small split of D1 as input. It can be observed that the difference between the memory used by one SparkBWA mapper considering regular and hybrid mode with 8 threads is only 4 GiB. It means an increase of about 30% in the total memory consumed, while the threads per mapper grows by a factor of 8.

So, taking into account that our experimental platform allows 22 containers per node with 11 GiB of maximum memory, SparkBWA in hybrid mode for this example could use all the 64 cores in the node, e.g., running 16 mappers and 4 threads/mapper. This is not the case of the regular mode, which only allows to use a maximum of 22 cores of the node. Therefore, the hybrid mode can be very useful in scenarios where the computing nodes consist of a high number of cores but, due to memory restrictions, only a few of them can be used.

**Fig 8:**
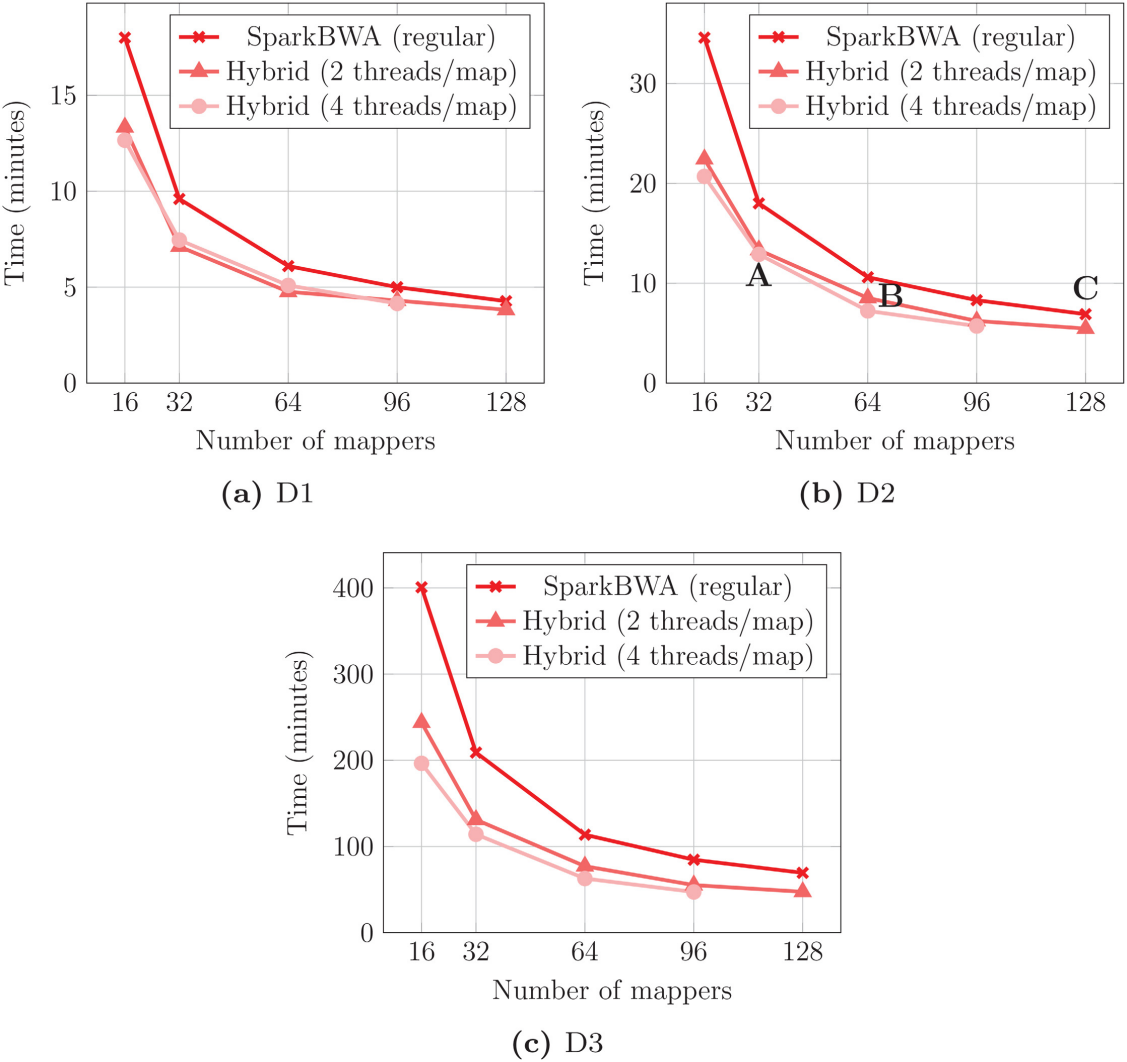
Execution times obtained by SparkBWA using regular and hybrid modes of operation for the BWA-MEM algorithm.

Next, we evaluate the performance of SparkBWA using both modes of operation. Experiments were conducted using the BWA-MEM algorithm and considering 2 and 4 threads per map process when hybrid mode is enabled. Performance results are shown in Fig 8 for all the datasets and using different number of mappers. There are no results for the 128 mappers with 4 threads/mapper case because it implies that 512 cores are necessary for an optimal execution, while our cluster only consists of 384 cores.

Several conclusions can be extracted from the performance results. SparkBWA shows a good scalability with the number of mappers, especially in the regular mode (that is, when each mapper is computed sequentially). Assuming the same number of mappers, more threads per mapper in the hybrid mode is only beneficial for the biggest dataset (D3). This behavior points out that the benefits of using more threads in the computations do not compensate the overhead caused by their synchronization.

On the other hand, considering the cores used in the computation (#threads × #mappers cores), we can observe that the regular mode performs better than the hybrid one. For instance, points A, B and C in Fig 8(b) were obtained using the same number of cores. SparkBWA in regular mode (point C) clearly outperforms the hybrid version. This behavior is observed in most of the cases. In this way, as we have indicated previously, SparkBWA hybrid mode should be the preferred option only in those cases where limitations in memory do not allow to use all the cores in each node.

Table 4 summarizes the results of SparkBWA in terms of performance for all the datasets. It shows the minimum time required by SparkBWA to perform the alignment on our hardware platform, the number of mappers used, the speed measured as the number of pairs aligned per second and also the corresponding speedup with respect to the sequential execution of BWA. The sequential times are respectively 258, 496 and 5,940 minutes for D1, D2 and D3. In the particular case of D3 it means more than 4 days of computation. It is worth noting that using SparkBWA this time was reduced to less than an hour reaching speedups higher than 125×.

**Table 4:** Summary of the performance results of SparkBWA.

Dataset	Mode of operation	No. of mappers	Time (minutes)	Pairs aligned/s	Speedup
D1	regular	128	4.3	46,512	60×
	hybrid (2 th/map)	128	3.8	52,632	67.9×
	hybrid (4 th/map)	96	4.1	48,780	62.9×
D2	regular	128	6.9	58,213	71.9×
	hybrid (2 th/map)	128	5.5	73,030	90.2×
	hybrid (4 th/map)	96	5.7	70,468	87.0×
D3	regular	128	69.4	23,727	85.6×
	hybrid (2 th/map)	128	47.5	34,667	125.0×
	hybrid (4 th/map)	96	47.3	34,813	126.2×

Finally, we verified the correctness of SparkBWA for regular and hybrid modes by comparing their output with the one generated by BWA (sequential version). We only found small differences in the mapping quality scores (mapq) on some uniquely mapped reads (i.e., reads with quality greater than zero). Therefore, the mapping coordinates are identical for all the cases considered. Differences affect from 0.06% to 1% of the total number of uniquely mapped reads. Small differences in the mapq scores are expected because the quality calculation depends on the insert size statistics, which are calculated on sample windows on the input stream of sequences. These sample windows are different for each read in BWA (sequential) and any other parallel implementation that splits the input into several pieces (SEAL, pBWA, Halvade, BWA-threaded version, SparkBWA, etc.). In this way, any parallel BWA-based aligner will obtain slightly different mapping quality scores with respect to the sequential version of BWA. For instance, SEAL reports differences on average in 0.5% of the uniquely mapped reads [21].

#### 5.2.3 Comparison to other aligners

Next, a performance comparison among different BWA-based aligners and SparkBWA is shown. The evaluated tools are enumerated in Table 3 together with their corresponding parallelization technology. Some of them take advantage of classical parallel paradigms, as Pthreads or MPI, while the others are based on big data technologies as Hadoop. All the experiments were performed using SparkBWA in regular mode. For comparison purposes all the graphs in this subsection include the corresponding results considering ideal speedup with respect to the sequential execution of BWA.

Two different algorithms for paired-end reads have been considered: BWA-backtrack and BWA-MEM. The evaluation of the BWA-backtrack algorithm was performed using the following aligners: pBWA, SEAL and SparkBWA. When paired reads are used as input data, BWA-backtrack consists of three phases. First, the sequence alignment must be performed for one of the input FASTQ files. Afterwards, the same action is applied to the other input file. Finally, a conversion to the SAM output format is performed using the results of the previous stages. SparkBWA and SEAL take care of the whole workflow in such a way that it is completely transparent to the user. Note that SEAL requires a preprocessing stage to prepare the input files, so this extra time was included in the measurements. On the other hand, pBWA requires to perform each phase of the BWA-backtrack algorithm independently despite they are executed in parallel. In this way, pBWA times were calculated as the sum of each phase time. No preprocessing is performed by pBWA.

**Fig 9:**
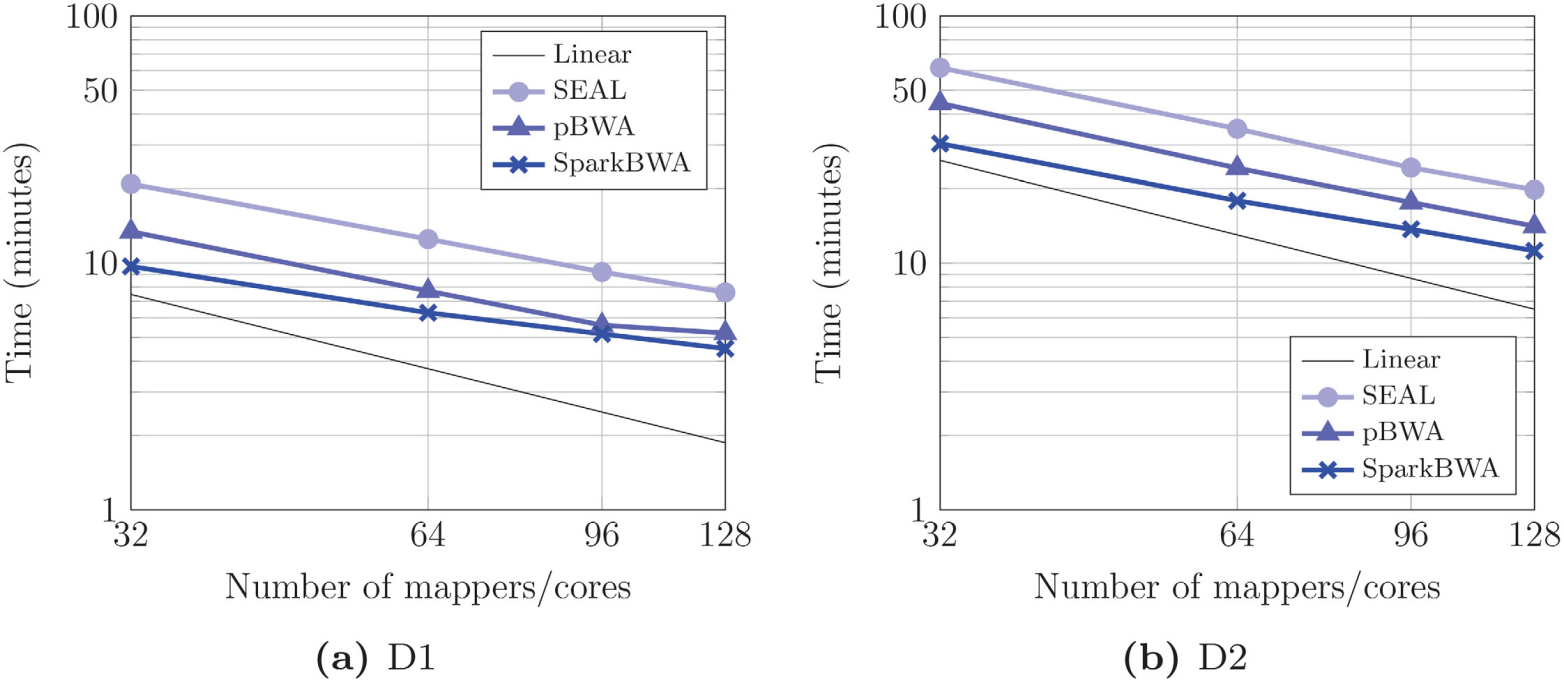
Execution times considering several BWA-based aligners running the BWA-backtrack algorithm (axes are in log scale).

As BWA-backtrack was especially designed for shorter reads (<100 bp), we have considered D1 as input dataset but, for completeness, D2 is also included in the comparison. Fig 9 shows the alignment times using different number of mappers. In this case, each map process uses one core, so both terms, mappers and cores, are equivalent. Results show that SparkBWA clearly outperforms SEAL and pBWA for all the cases. As we have mentioned previously, SEAL times include the overhead caused by the preprocessing phase which takes on average about 1.9 and 2.9 minutes for D1 and D2 respectively. This overhead has a large impact on performance, especially for the smallest dataset.

**Fig 10:**
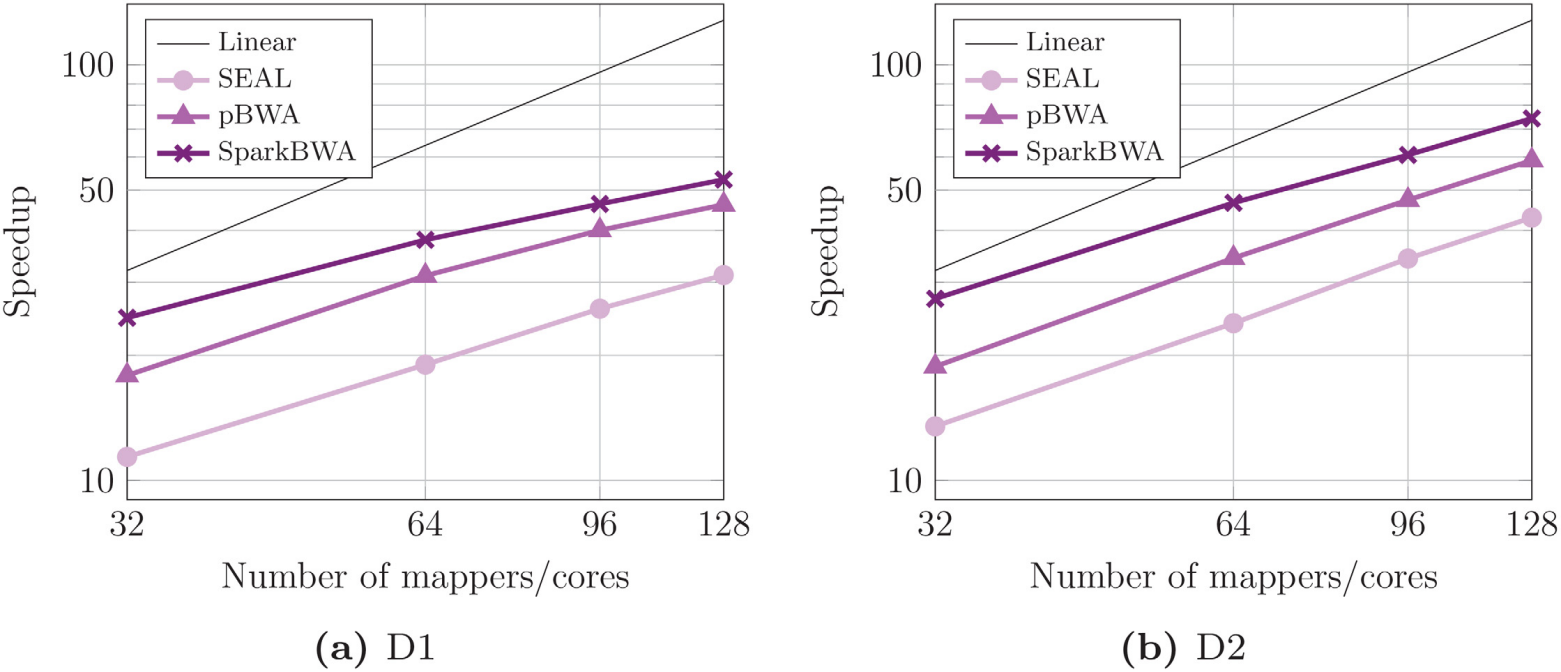
Speedup considering several BWA-based aligners running the BWA-backtrack algorithm (axes are in log scale).

The corresponding speedups obtained by the aligners for BWA-backtrack are displayed in Fig 10. As reference we have used the BWA sequential time. Results confirm the good behavior of SparkBWA with respect to SEAL and pBWA. For instance, SparkBWA reaches speedups up to 57× and 77× for D1 and D2 respectively. The maximum speedups achieved by SEAL are only about 31× and 42×, while the corresponding values for pBWA are 46× and 59×. In this way, SparkBWA is on average 1.9× and 1.4× faster than SEAL and pBWA respectively.

**Fig 11:**
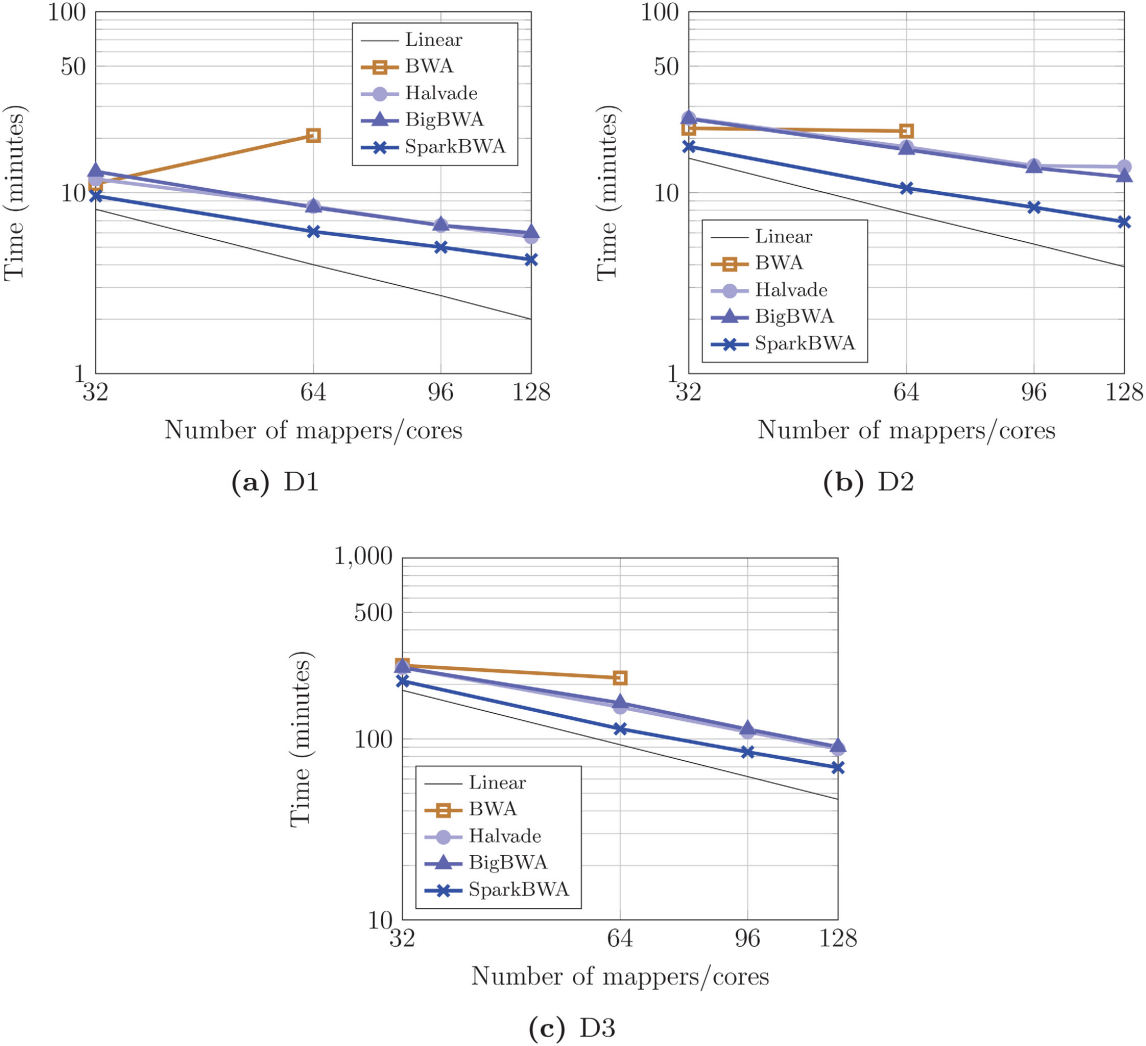
Execution times considering several BWA-based aligners running the BWA-MEM algorithm (axes are in log scale).

Finally, the BWA-MEM algorithm is evaluated considering the following tools: BWA, BigBWA, Halvade, and SparkBWA. Fig 11 shows the corresponding execution times for all the datasets varying the number of mappers (cores). BWA uses Pthreads in order to parallelize the alignment process, so it can only be executed on a single cluster node (64 cores). Both BigBWA and Halvade are based on Hadoop, and they require a preprocessing stage to prepare the input data for the alignment process. BigBWA requires, on average, 2.4, 5.8 and 23.6 minutes to preprocess each dataset, whereas Halvade spends 1.8, 6.6 and 22.7 minutes, respectively. Preprocessing is carried out sequentially for BigBWA, while Halvade is able to perform it in parallel. This overhead does not depend on the number of mappers used in the computations. For comparison fairness, the overhead of this phase is included in the corresponding execution times of both tools, since times for BWA and SparkBWA encompass the whole alignment process.

Performance results show that BWA is competitive with respect to Hadoop-based tools (BigBWA and Halvade) when 32 mappers are used, but its scalability is very poor. Using more threads in the computations do not compensate the overhead caused by their synchronization unless the dataset was big enough. BigBWA and Halvade show a better overall performance with respect to BWA. Both tools behave in a similar way, and differences in their performance are small. Finally, SparkBWA outperforms all the considered tools. In order to illustrate the benefits of our proposal it is worth noting that, for example, SparkBWA is on average 1.5× faster than BigBWA and Halvade when using 128 mappers, and 2.5× with respect to BWA considering 64 mappers.

**Fig 12:**
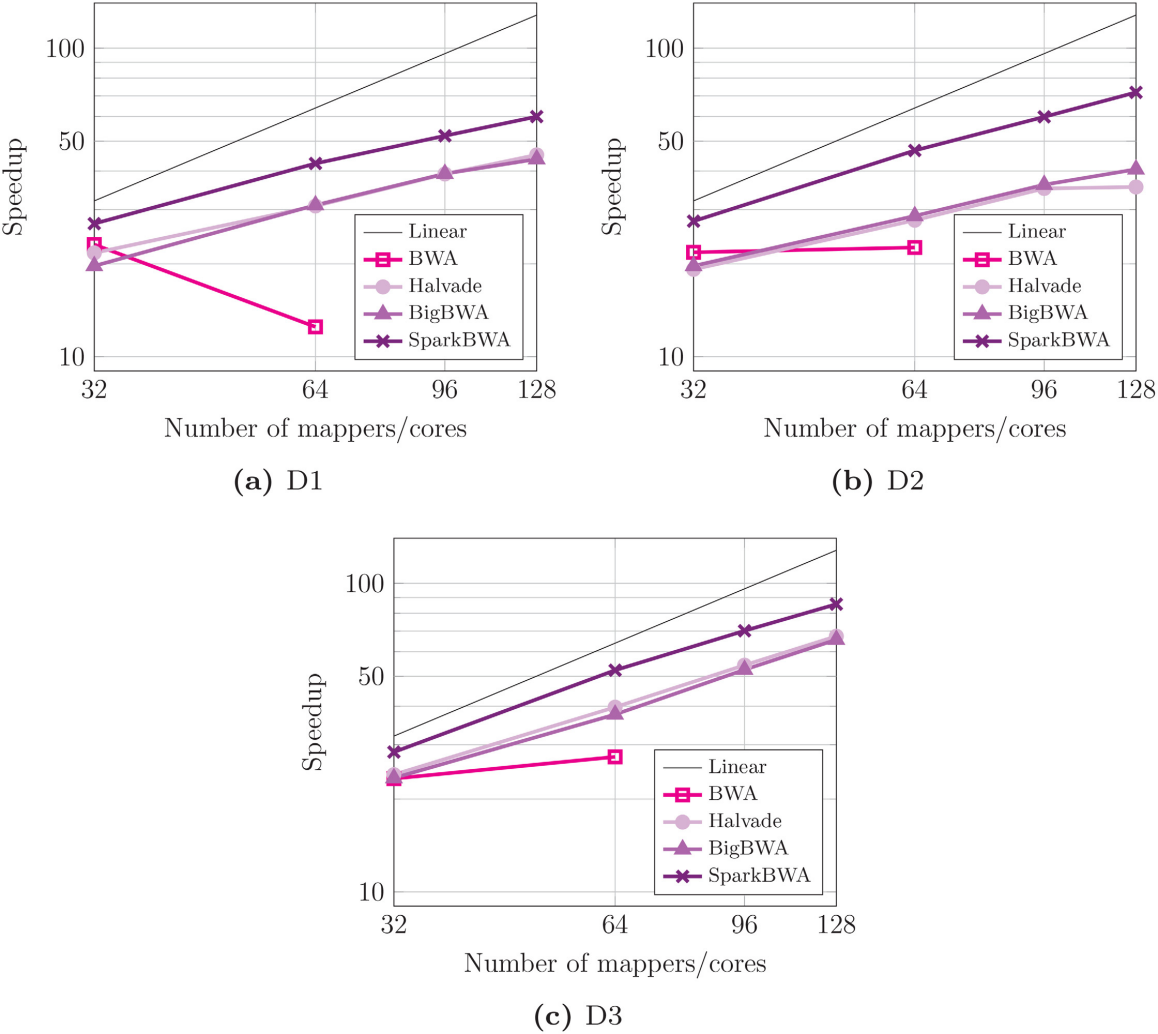
Speedup considering several BWA-based aligners running the BWA-MEM algorithm (axes are in log scale).

Performance results in terms of speedup with respect to the sequential execution of BWA are shown in Fig 12. The scalability problems of BWA are clearly revealed in the graphs. Hadoop-based tools show a better scalability but it is not enough to get closer to SparkBWA. The average speedup is respectively 50× and 49.2× for BigBWA and Halvade using 128 workers. This value increases up to 72.5× for SparkBWA. Note that the scalability of SparkBWA is especially good when considering the biggest dataset (Fig 12(c)), reaching a maximum speedup of 85.6×. In other words, the parallel efficiency is 0.67.

In this way, SparkBWA has proven to be very consistent in all the scenarios considered, improving the results obtained by other state of the art BWA-based aligners. In addition, we must highlight that SparkBWA behaves better as the size of the dataset increases.

## 6 Conclusions

In this work we introduce SparkBWA, a new tool that exploits the capabilities of a Big Data technology as Apache Spark to boost the performance of the Burrows-Wheeler Aligner (BWA), which is a very popular software for mapping DNA sequence reads to a large reference genome. BWA consists of several algorithms especially tuned to deal with the alignment of short reads. SparkBWA was designed in such a way that no modifications to the original BWA source code are required. In this way, SparkBWA keeps the compatibility with any BWA software release, future or legacy.

The behavior of SparkBWA was evaluated in terms of performance, scalability and memory consumption. In addition, a thorough comparison between SparkBWA and several state of the art BWA-based aligners was performed. Those tools take advantage of different parallel approaches as Pthreads, MPI, and Hadoop to improve the performance of BWA. The evaluation shows that when considering the algorithm to align shorter reads (BWA-backtrack), SparkBWA is on average 1.9× and 1.4× faster than SEAL and pBWA. For longer reads and the BWA-MEM algorithm, the average speedup achieved by SparkBWA with respect to BigBWA and Halvade tools is 1.4×.

Finally, it is worth noting that most of the next-generation sequencing (NGS) professionals are not experts in Big Data or High Performance Computing. For this reason, in order to make SparkBWA more suitable for these professionals, an easy and flexible API is provided which will facilitate the adoption of the new tool by the community. This API allows to manage the sequence alignment process from the Apache Spark shell, hiding all the computational details to the users.

The source code of SparkBWA is publicly available at the GitHub repository (https://github.com/citiususc/SparkBWA).
